# Prognostic DNA methylation markers for sporadic colorectal cancer: a systematic review

**DOI:** 10.1186/s13148-018-0461-8

**Published:** 2018-03-14

**Authors:** Muriel X. G. Draht, Danny Goudkade, Alexander Koch, Heike I. Grabsch, Matty P. Weijenberg, Manon van Engeland, Veerle Melotte, Kim M. Smits

**Affiliations:** 1 Department of Pathology, GROW - School for Oncology and Developmental Biology, Maastricht University Medical Center, Maastricht, The Netherlands; 2 Pathology and Tumour Biology, Leeds Institute of Cancer and Pathology, University of Leeds, Leeds, UK; 3 Department of Epidemiology, GROW - School for Oncology and Developmental Biology, Maastricht University Medical Center, Maastricht, The Netherlands; 4 Department of Clinical Genetics, University of Rotterdam, Rotterdam, The Netherlands

**Keywords:** Biomarker, DNA methylation, Methylation marker, Colorectal cancer, Colon cancer, Prognosis, Survival, Patient outcome, REMARK

## Abstract

**Background:**

Biomarkers that can predict the prognosis of colorectal cancer (CRC) patients and that can stratify high-risk early stage patients from low-risk early stage patients are urgently needed for better management of CRC. During the last decades, a large variety of prognostic DNA methylation markers has been published in the literature. However, to date, none of these markers are used in clinical practice.

**Methods:**

To obtain an overview of the number of published prognostic methylation markers for CRC, the number of markers that was validated independently, and the current level of evidence (LoE), we conducted a systematic review of PubMed, EMBASE, and MEDLINE. In addition, we scored studies based on the REMARK guidelines that were established in order to attain more transparency and complete reporting of prognostic biomarker studies. Eighty-three studies reporting on 123 methylation markers fulfilled the study entry criteria and were scored according to REMARK.

**Results:**

Sixty-three studies investigated single methylation markers, whereas 20 studies reported combinations of methylation markers. We observed substantial variation regarding the reporting of sample sizes and patient characteristics, statistical analyses, and methodology. The median (range) REMARK score for the studies was 10.7 points (4.5 to 17.5) out of a maximum of 20 possible points. The median REMARK score was lower in studies, which reported a *p* value below 0.05 versus those, which did not (*p* = 0.005). A borderline statistically significant association was observed between the reported *p* value of the survival analysis and the size of the study population (*p* = 0.051). Only 23 out of 123 markers (17%) were investigated in two or more study series. For 12 markers, and two multimarker panels, consistent results were reported in two or more study series. For four markers, the current LoE is level II, for all other markers, the LoE is lower.

**Conclusion:**

This systematic review reflects that adequate reporting according to REMARK and validation of prognostic methylation markers is absent in the majority of CRC methylation marker studies. However, this systematic review provides a comprehensive overview of published prognostic methylation markers for CRC and highlights the most promising markers that have been published in the last two decades.

**Electronic supplementary material:**

The online version of this article (10.1186/s13148-018-0461-8) contains supplementary material, which is available to authorized users.

## Background

Colorectal cancer (CRC) is the third most common form of cancer and accounts for more than 500,000 deaths worldwide each year [1]. Overall, the prognosis of CRC patients is poor with about half of all diagnosed patients dying as a result of recurrence, metastasized disease, or co-morbidities [2]. CRC often develops without symptoms until it has reached an advanced stage. Prognostic markers, which can predict the prognosis of CRC patients and which can stratify high-risk early stage patients from low-risk early stage patients are urgently needed for better management of CRC. Evidence-based results regarding prognostic markers are therefore essential for better patient management.

CRC patient survival is highly dependent on the tumor stage at the time of diagnosis. Therefore, the tumor-node-metastasis (TNM) staging system is the gold standard to determine the prognosis of a CRC patient [3, 4]. In addition, clinical markers, such as poor tumor differentiation, vascular, and/or perineural invasion, as well as molecular markers, such as microsatellite instability (MSI) status and *KRAS* or *BRAF* mutation status can be used [5, 6]. Since in many years, a vast amount of epigenetic biomarkers have been identified and described as promising cancer biomarkers in the scientific literature [7–10]. However, to date, only a few biomarkers have been validated for clinical use [11, 12]. CRC, in particular, has often been the topic of epigenetic biomarker research, leading to the identification of methylation markers for early detection of CRC, prediction of prognosis, and/or treatment response [8, 13–16]. At the moment some methylation markers for early detection of CRC (such as *SEPT9*, *NDRG4*, and *BMP3*) have been incorporated in the FDA-approved commercial tests, Epi proColon® and Cologuard, respectively [17, 18]. However, for prognostic or predictive purposes, no methylation marker for colon and/or rectal cancer has made the translation to a clinically applicable biomarker. The reasons for the lack of translation of biomarkers, prognostic, or other, into clinical practice, have already been recognized previously, with many different research groups providing possible explanations and/or solutions for these problems, such as poorly selected biospecimens, not-clinically relevant sample series, and underpowered sample series, as well as lack of validation and reproducibility of the biomarker assay [19–24]. In 2005, the REporting recommendations for tumor MARKer prognostic studies (REMARK) guidelines were published in an effort to improve the reporting of biomarker studies and subsequently increase the number of prognostic biomarkers that can be used in clinical practice [25]. Nevertheless, there is evidence that adherence to REMARK is still suboptimal [26, 27].

A comprehensive overview of potentially promising prognostic epigenetic biomarkers for CRC is lacking. Furthermore, the current amount of available information only leads to more confusion instead of contributing to answering the question, which biomarker should be further developed for translation. Here, we provide a comprehensive overview of the currently available evidence on prognostic DNA methylation markers for CRC and review the quality of these studies using the REMARK guidelines as a tool.

## Methods

### Search strategy and study eligibility

**Fig. 1:**
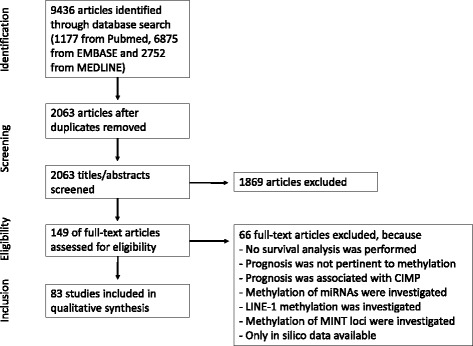
Flowchart of the study identification process. A total of 83 studies were selected for qualitative assessment

A literature review was performed covering English language articles in PubMed, EMBASE, and MEDLINE until May 2017 using the following search terms: DNA methylation, biomarker, cancer, colon, colorectum, colorectal, survival, patients outcome, prognosis (Additional file 1). Published studies were eligible to be included in our analysis if colon, rectal, or colorectal cancer patient prognosis was analyzed stratifying patients by methylation status of the marker. Only original articles (no reviews, editorials, conference abstracts, etc.) were considered (Fig. 1). Studies were included if overall survival (OS), disease-specific survival (DSS), disease-free survival (DFS), recurrence-free survival (RFS), or any other endpoint were reported and if results were presented as Kaplan-Meier plots, relative risks, or hazard ratios (HRs) with corresponding 95% confidence intervals (95% CI). We did not restrict our search to specific patient characteristics (such as age group, sex, ethnicity, and tumor type). Studies were excluded if the tumor was hereditary; prognosis was not analyzed by one of the abovementioned methods; studies were focusing on the prognostic influence of the CpG island methylator phenotype (CIMP and microsatellite instability (including MINT loci)), as this has been the topic of multiple systematic reviews of our and other research groups [28–32]; and studies were on methylated miRNAs and LINE-1, as our focus in this review was directed to CpG islands of protein-coding genes.

### Data extraction

Data extraction was performed by two independent researchers (MD and DG) using a standardized data registration form in which the following items were recorded: marker of study, sample size, cancer type (colon, rectum, or CRC), sample type (primary tissue, serum, mucosa, blood, lymph node tissue, peritoneal lavage, or stool), stage (tumor-node-metastasis (TNM) staging, according to editions mentioned in original paper, or Dukes’ staging), study design, year of collection of samples, number of patients in survival analyses, endpoints, subgroup analysis, *p* value, and hazard ratio (HR) with corresponding confidence interval. This systematic review was conducted according to the Preferred Reporting Items for Systematic Reviews and Meta-Analyses (PRISMA) statement, where applicable [33].

### Quality assessment

Eligible studies were scored (MD and DG) based on the REMARK criteria [34], which summarizes 20 items for good reporting of a prognostic biomarker study (Additional file 2). In case of complete reporting according to the guidelines, a study was given 1 point, in case of incomplete reporting, a study was given 0.5 points, and in case of lack of reporting any aspect of the guideline item, a study was given 0 points. The maximum score was 20 points (all items adequately reported). Interobserver variation of scores was solved by mutual consensus. The risk of potential bias and confounders was analyzed per study using the information obtained with the REMARK scores. If a study obtained ≥ 1.5 points for REMARK criterion #2 (“patient characteristics”) and #6 (“sample selection and follow-up”), the risk of selection bias was low. In case of less than 1.5 points, the risk of potential selection bias is increased. Bias regarding the assay method (measurement bias) was assessed similarly using REMARK criteria #5 (“assay method”) and #11 (“handling of marker values”). The risk of bias regarding outcome assessment (measurement bias) was scored based on REMARK criterion #7 (“clinical endpoint definition”). In case of complete reporting (score = 1), the risk of bias is low, as compared to partial or lack of reporting, which increases a potential risk of bias. The presence of potential confounding factors was assessed using REMARK criterion #16 (“multivariable analysis”). In case of 1 point, the risk of confounding factors is lower as compared to studies that did not perform or report a multivariable analysis. In order to investigate whether the REMARK score or the total number of patients included in the survival analysis correlates with the reported significance of the marker (*p* value), we performed a regression analysis and determined the Pearson’s correlation coefficient (*r*). We compared the REMARK scores of studies reporting a significant finding versus studies reporting a non-significant finding using a Mann-Whitney test. We used the statistical programming language R (version 3.3.1) to perform all analyses.

### Forest plots

We prepared forest plots for methylation markers that were investigated in two or more study series. If available, we reported HRs for overall and subgroup analysis, such as single TNM stages or mutation status. Univariate HRs were used, unless multivariate HRs were available. If multiple HRs were available, the most adjusted HR was depicted in the plot. In order to give a complete overview, *p* values are depicted in the forest plot, if only Kaplan-Meier results were available. We used the statistical programming language R (version 3.3.1) to perform all analyses and generate the figures.

### Level of evidence

The level of evidence (LoE) can be determined using an evidence-ranking scheme such as GRADE [35] or the OCEBM levels [36] in which level I represents definitive evidence, level IV represents (very) weak evidence, and the remaining levels a degree in between. Even though these rankings do not provide a definitive judgment on the quality of the provided evidence, they do offer a valuable indication. To give an overview of the current evidence on prognostic epigenetic biomarkers in CRC, we classified a LoE to each marker, or marker panel, investigated in two or more independent study series, using a ranking scheme adapted for biomarkers [37] and the OCEBM schemes [36].

## Results

### Eligible studies

We initially identified 2063 studies for potential inclusion using our search strategy (Additional file 1). We excluded 1869 studies mainly because they were either not original studies or not relevant to prognosis or colorectal cancer, colon cancer, or rectal cancer. We checked full-text articles of the remaining 194 studies, of which 66 were excluded, for prognosis was not pertinent to methylation, prognosis was associated with CIMP, methylation of non-protein-coding genes was investigated (miRNAs, LINE-1, MINT loci), or only in silico data were described (Fig. 1). A total of 83 studies were included in this systematic review.

### Study characteristics

Study characteristics are summarized in Additional file 3. Sixty-three (76%) studies reported results from single methylation markers, and 20 (24%) studies included multiple markers. In total, 123 different methylation markers were investigated (Additional file 3). Studies were published between 1999 and 2017. Median (range) sample size was 127 patients (30 to 1105 patients). Seventy-four (89%) studies investigated CRC, six (7%) studies investigated colon cancer only, and three (4%) studies investigated rectal cancer only. Sixty-eight (82%) studies used formalin-fixed or fresh-frozen primary tissue for biomarker analyses, 10 (12%) studies used blood (serum or plasma), two (3%) studies used normal mucosa, one study (1%) used peritoneal lavage fluid, and in one study (1%), the tissue used in the analyses was not specified. Thirteen (16%) studies included patients with the same single TNM (or Dukes) stage, 68 (82%) studies included patients with two or more disease stages. For two (3%) studies, the TNM (or Dukes) stage of the included patients was not specified. Eighty-two studies (99%) used Cox proportional hazard analyses, Kaplan-Meier plots, or both to assess the relation with overall, regression-free, or disease-specific survival. For one study (1%), the statistical method was not described.

### Quality assessment

**Fig. 2:**
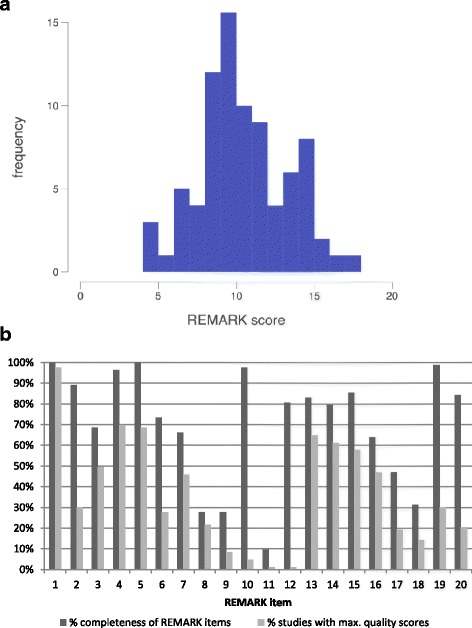
Quality assessment of methylation marker studies. **a** Histogram depicting the REMARK score distribution for all studies included in the analysis (mean REMARK score = 10.711, standard deviation = 2.820). **b** Histogram showing the completeness of reported REMARK items

We evaluated studies according to the REMARK checklist and assigned a score between 0 and 20 to each study (Additional file 4). The scores ranged from 4.5 points to 17.5 points with a median score of 10.7 (Fig. 2a). Among the 83 studies that were scored according to the REMARK criteria, we observed a large variation in the amount of information given for the specific criterion. For only two criteria (#1“state marker, objectives, and hypotheses” and #5 “specify assay details”), complete or partial information was given for all studies. For the other criteria, these numbers ranged from 10% (#11 “specify marker values in analyses and discuss cut-off points”) to 99% (#19 “interpretation of results and study limitations”) (Fig. 2b). Full quality scores could only be given for one REMARK criterion (#1 “state marker, objectives, and hypotheses”) for the majority of the studies (98%). For all other criteria, the percentage of studies obtaining full quality scores ranged from 1% (#11 “specify marker values in analyses and discuss cutoff points”) to 70% (#4 “describe biological material”) (Fig. 2b). Almost none of the studies sufficiently addressed how marker values were handled in the analyses or presented cutoffs (10%). Less than half of the studies provided complete or partial information on candidate markers initially considered for the study (28%), reported a rationale for sample size (28%), reported estimated effects with corresponding confidence intervals of the marker and other prognostic variables in the analyses (47%), or reported further investigations such as checking assumptions of proportional hazards (31%). A complete overview of the REMARK scores for the different studies per REMARK criterion is presented in Additional file 4. The risk of bias of each included study is summarized in Additional file 5.

**Fig. 3:**
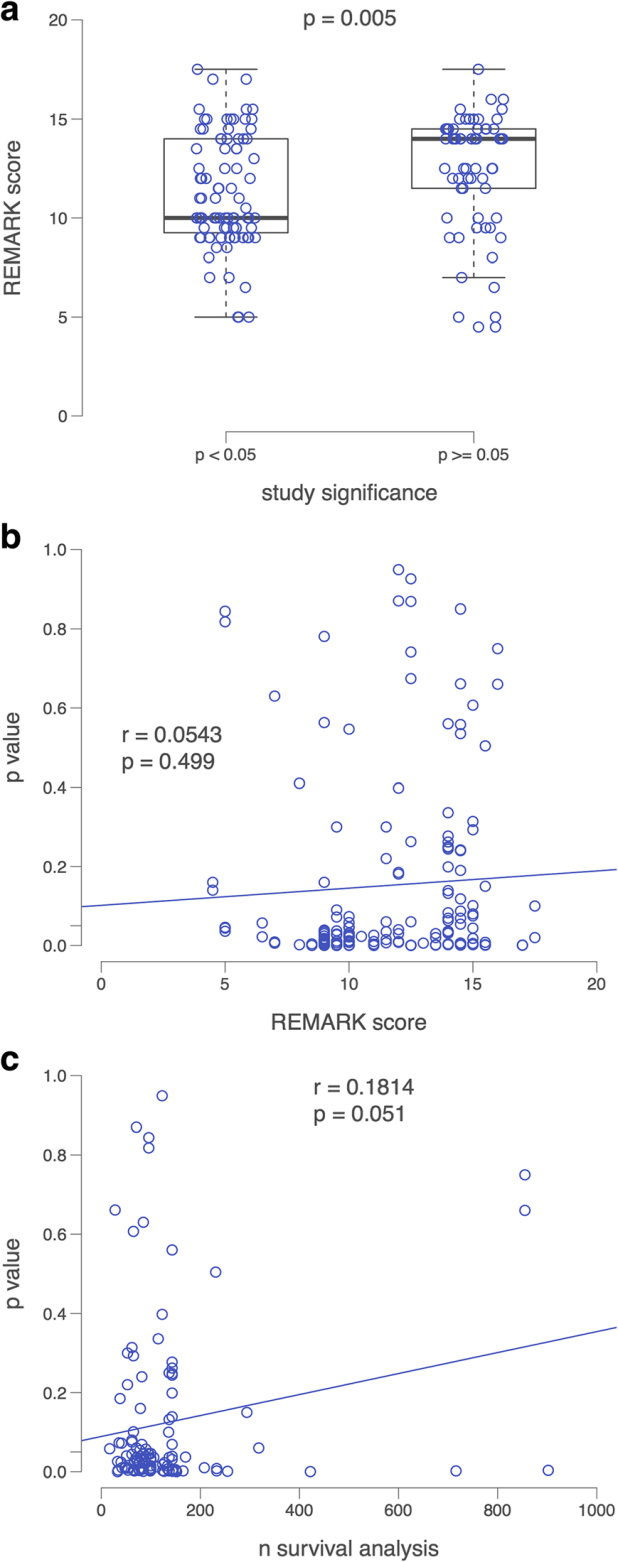
Association between REMARK score and significance level of studies. **a** Box plot comparing the REMARK score between studies that reported statistically significant findings versus studies that did not report statistically significant findings (Mann-Whitney test, *p* = 0.005). **b** Dot plot showing that there was, however no significant correlation between the REMARK score and the reported *p* values (Pearson’s correlation coefficient = 0.0543, *p* value = 0.499). **c** Dot plot depicting that for the reported survival analyses, we found a stronger, but still statistically not significant correlation between the number of patients used and the reported *p* values (Pearson’s correlation coefficient = 0.1814, *p* value = 0.051)

Since it is more likely that studies reporting statistically significant results get published [38, 39], we were interested whether there is an association between REMARK score and reported *p* value and whether inadequately reported studies tend to more frequently report significant results. Although REMARK scores varied between the individual studies, *p* values < 0.05 for the association between the methylation marker and prognosis were more often reported in studies with lower REMARK scores, as compared to studies with average to high REMARK scores (Fig. 3a, *p* = 0.005), although this was not seen in the Pearson’s correlation coefficient (Fig. 3b; *r* = 0.0543, *p* = 0.499). Whereas almost half (46%) of the 83 selected studies exclusively reported significant results, 24% of all studies reported non-significant methylation marker results and 30% described statistically significant, as well as non-significant results. Often, methylation markers were reported in small study populations (median *n* = 127.5), increasing the possibility that reported prognostic effects cannot be validated in other study populations. Therefore, we were interested whether there is an association between the reported *p* value and the number of patients included in the survival analysis. A borderline statistically significant correlation was observed between the reported *p* value of the survival analysis and the size of the study population (*n*) (Fig. 3c; *p* = 0.051).

### Prognostic marker findings

**Fig. 4:**
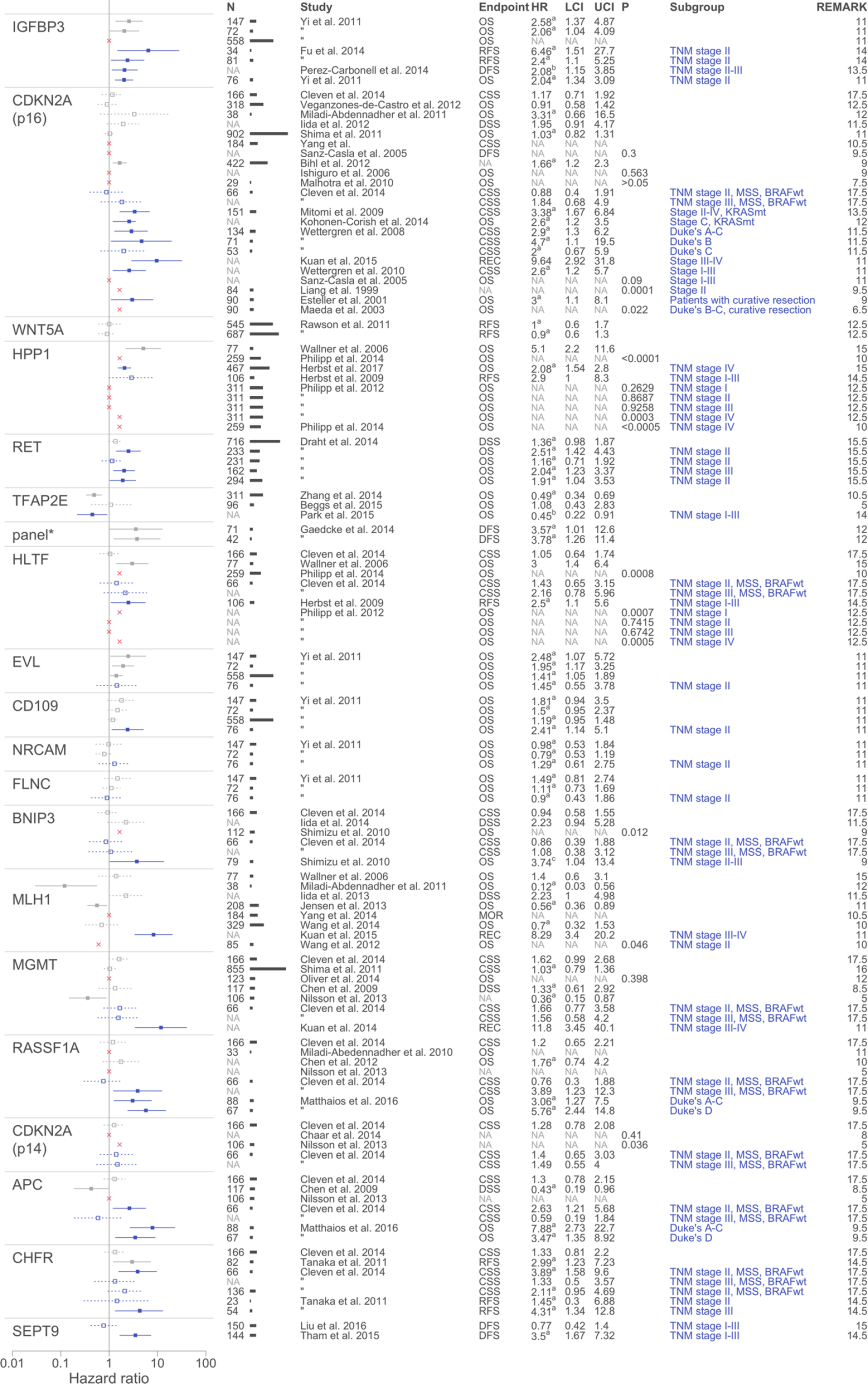
Forest plots of reported methylation markers in colorectal cancer studies. Forest plots were prepared for methylation markers that were reported in two or more publications or study populations. The hazard ratios (HR) are sorted according to the REMARK score. HRs with a statistically significant association are depicted with a solid line; HRs of reported markers with no significant association are depicted with a dotted line; HRs of subgroup analyses are depicted in blue. Univariate HRs and confidence intervals (CI) are reported unless multivariate HRs were available (**a**). As for *IGFBP3* and *TFAP2E* the HRs of the study of Perez-Carbonell et al. [90] and Zhang et al. [117], respectively, were both associated with worse survival. For this figure, the HR was reversed for visualization purposes (**b**). A multivariate HR for BNIP3 methylation was available in the study of Shimizu et al., however was not statistically significant (**c**)

Additional file 3 shows the impact of methylation markers on prognosis in the included studies [40–122]. The majority of markers were investigated in a single study without any internal or external validation. As unvalidated results are at higher risk to represent chance findings, validation in at least one independent population is needed to draw any conclusion, even preliminary, for these markers. Therefore, we only prepared forest plots for methylation markers that were investigated by two or more studies and/or where internal validation in an independent series was performed (i.e., *IGFBP3*, *CDKN2A* (*p16*), *WNT5a*, *HPP1*, *RET*, *TFPA2E*, *HLTF*, *EVL, CD109, NRCAM, FLNC*, *BNIP3*, *MLH1*, *MGMT*, *RASSF1A*, *CDKN2A* (*p14*), *APC*, *CHFR*, *SEPT9*, and one multimarker panel; Fig. 4).

Overall, studies assessing *IGFBP3* methylation showed similar correlations with a poor prognosis in CRC patients. Yi et al. firstly investigated *IGFBP3* hypermethylation as a prognostic marker in three different study populations [48]. Although a significant association was found with poorer OS in the two smaller populations (*n* = 147, *n* = 72; HR 2.58 95% CI 1.37–4.87, HR 2.06 95% CI 1.04–4.09, respectively), no association was found in a cohort of 558 patients (data not shown in original article). In a subgroup analysis, *IGFBP3* hypermethylation was found to be a prognostic factor in three independent studies, all focusing on TNM stages II–III, even though every study used a different endpoint (RFS, DFS, and OS) (HR 6.46 95% CI 1.51–27.70; HR 2.40 95% CI 1.10–5.25; HR 0.48 95% CI 0.26–0.87; HR 2.04 95% CI 1.34–3.09) [48, 89, 90].

*CDKN2A* (*p16*) was by far the most often studied biomarker, with ten studies in TNM stage I-IV [53, 57, 59–61, 63–65, 70, 73] and 13 studies in different subgroups [50–53, 55, 56, 58, 62, 69, 73]. Only one study in TNM stages I–IV showed a statistically significant association between *CDKN2A* methylation and outcome (HR 1.66 95% CI 1.2–2.3) [60]. Studies including subgroup analyses however showed statistically significant associations between *CDKN2A* methylation and a poor prognosis in several different subgroups. The studies by Kohonen-Corish et al., Wettergren et al. 2008, and Esteller et al. 2001 in TNM stages I–III and Dukes’ stage A–C patients describe a statistically significant association (HR 2.60 95% CI 1.20–5.70; HR 2.90 95% CI 1.30–6.20; HR 3.00 95% CI 1.10–8.10, respectively) [51, 55, 62], although the study of Sanz-Casla et al. could not confirm this (*p* = 0.09) [53]. The studies of Liang et al. [50], Wettergren et al. [55], Mitomi et al. [58], and Maeda et al. [52], focusing on TNM stage II or Dukes’ B patients alone or in a larger subgroup of TNM stages II–IV or Dukes’ B–C patients, also show statistically significant associations with a poor prognosis (*p* = 0.0001; HR 4.70 95% CI 1.10–19.50; HR 3.38 95% CI 1.67–6.84; *p* = 0.022, respectively). This could not be confirmed by the study of Cleven et al.*,* which was conducted in TNM stage II, microsatellite stable (MSS), and *BRAF*wt patients [73]. A significant association with a poor prognosis was also reported for Dukes’ C patients with mutated *KRAS* (HR 2.60 95% CI 1.20–3.50) [62], but not in another study focusing on Dukes’ C patients or TNM stage III, MSS, and *BRAF*wt patients [73].

Methylation of *WNT5a* was only studied in one publication. Rawson et al. investigated methylation of *WNT5a* in two large independent series, showing no association between methylation of *WNT5a* and prognosis (HR 1.0 95% CI 0.6–1.7; HR 0.9 95% CI 0.6–1.3) [122].

Four studies reported on the association between *HPP1* hypermethylation and prognosis with conflicting results [78–81]. In TNM stages I–IV, a statistically significant association was shown with OS (HR 5.10 95% CI 2.20–11.60 and Kaplan-Meier *p* value < 0.0001) [78, 81]. Subgroup analyses of the studies by Philipp et al. showed a statistically significant association between OS and *HPP1* hypermethylation in TNM stage IV only (Kaplan-Meier *p* value 0.0003 and < 0.0001, respectively) [80, 81]. The study by Herbst et al. only showed a borderline statistically significant association with RFS (HR 2.90 95% CI 1.00–8.30) [79]. A recent prospective study by Herbst et al., including 467 TNM stage IV patients, reported a significant poorer OS in patients with *HPP1* methylation (HR 1.86 95% CI 1.37–2.53 for univariate analysis) [82]. After one administration with combination chemotherapy, a multivariate analysis still predicted a poorer outcome for *HPP1* methylated patients (HR 2.08 95% CI 1.54–2.80).

Methylation of *RET* was studied in three independent patient series reported in one publication [107]. While there was no association with disease-specific survival in the total population of TNM stage I–IV patients, a significant association with poorer OS was found in two TNM stage II patient series (HR 2.51 95% CI 1.42–4.43; HR 1.91 95% CI 1.04–3.53) and one TNM stage III patient series (HR 2.04 95%-CI 1.23–3.37).

Hyper- as well as hypomethylation of *TFAP2E* was investigated in three studies, of which two studies strikingly reported similar associations between hyper- and hypomethylation and prognosis. The study by Zhang et al. assessed the influence of *TFAP2E* hypermethylation on prognosis in TNM stage I-IV patients, showing a significant association between *TFAP2E* hypermethylation and a favorable prognosis (HR 0.49 95%-CI 0.34–0.69) [117]. A subgroup analysis by Park et al. of TNM stage I-III patients confirmed this association (HR 2.24 95%-CI 1.10–4.56; HR converted for figure) [118]. In the study of Beggs et al. hypermethylation of *TFAP2E* was not associated with survival (HR 10.8 95%-CI 1.08–2.83), however a survival benefit was found in patients with *TFAP2E* hypomethylation (HR 0.34 95%-CI 0.12–0.97) [119].

Methylation of *HLTF* was investigated in five studies with conflicting results. A statistically significant association between *HLTF* hypermethylation and OS in TNM stage I-IV CRC patients was suggested by Wallner et al. and Philipp et al. (HR 3.0 95% CI 1.40–6.40 and Kaplan-Meier *p* value 0.0008) [78, 81], whereas the study of Cleven et al., using CSS as an endpoint, could not confirm this finding (HR 1.05 95%-CI 0.64–1.74) [73]. Subgroup analyses of TNM stage II and III patients showed no statistically significant association between *HLTF* hypermethylation and OS or CSS [73, 80]. In TNM stages I and IV, and I-III however, a statistically significant association was observed between *HLTF* hypermethylation and OS or RFS (Kaplan-Meier *p*-values 0.0007 and 0.0005, and HR 2.50 95%-CI 1.10–5.60, respectively) [79, 80].

Next to *IGFBP3*, Yi et al. also investigated four other genes (*EVL*, *CD109*, *NRCAM*, and *FLNC*) in three independent populations [48]. However, only methylation of *EVL* appeared to have a significant association with worse OS in TNM stage I–IV colon cancer patients (HR 2.48 95% CI 1.07–5.72; HR 1.95 95% CI 1.17–3.25; HR 1.41 95% CI 1.05–1.89). Methylation of *CD109* was only significantly associated with poorer OS in 76 TNM stage II patients (HR 2.41 95% CI 1.14–5.1). Yi et al. also investigated combinations of these markers with and without *IGFBP3*; however, significant results were only reported for the two smaller populations of TNM stage I–IV colon cancer patients (*n* = 147, *n* = 72) [48].

Conflicting results were also found for *BNIP3* methylation, which was studied in three different studies [47, 65, 73]. In overall univariate analyses, *BNIP3* hypermethylation appeared to be statistically significantly associated with a poorer survival in the study by Shimizu et al. (Kaplan-Meier *p* value 0.012), whereas the other two studies did not report a statistically significant association with poor prognosis (HR 0.94 95% CI 0.58–1.55 and HR 2.23 95% CI 0.94–5.28). Subgroup analyses of *BNIP3* methylation showed a statistically significant association with poorer OS in one of two studies (HR 3.74 95% CI 1.04–13.43) [47] but not in the other (TNM stages II and III, MSS, *BRAF*wt patients HR 0.86 95% CI 0.39–1.88, and HR 1.08 95% CI 0.38–3.12, respectively) [73].

Out of six studies assessing the association between *MLH1* methylation and prognosis in TNM stages I–IV, three studies showed statistically significant results; however, two showed a better prognosis (HR 0.12 95% CI 0.03–0.56 and HR 0.56 95% CI 0.36–0.89, respectively) [64, 83], while the study by Iida et al. showed a borderline significant association with a poorer DSS (HR 2.23 95% CI 1.00–4.98) [65]. The studies by Wallner et al., Yang et al., and Wang et al. showed no association [70, 78, 84]. Subgroup analyses by Kuan et al. and Wang et al. also show these conflicting results with one reporting a statistically significant high recurrence risk (HR 8.29 95% CI 3.40–20.22) [69], while the other report a better OS when *MLH1* is methylated (*p* = 0.046) [49].

For *MGMT* promoter hypermethylation, four studies showed no association with prognosis in TNM stages I–IV [43, 73, 94, 95], while the study of Nilsson et al. suggested an association between *MGMT* methylation and a better prognosis (HR 0.36 95% CI 0.15–0.87) [105]. Subgroup analyses in TNM stages II and III showed no association with *MGMT* in the study of Cleven et al. [73]. Strikingly, a study by Kuan et al. focused on recurrence and showed a strong association between recurrence and *MGMT* methylation in TNM stages III–IV (HR 11.83 95% CI 3.45–40.12) [69].

*RASSF1A* methylation was studied in five different studies; however, a statistically significant association was only found in TNM stage III patients (HR 3.89 95% CI 1.23–12.30) [73]. Four studies in TNM stages I–IV and another subgroup analysis on TNM stage II did not show any association between *RASSF1* and prognosis [73, 103–105]. Matthaios et al. investigated *RASSF1* methylation in Dukes’ A–C and in Dukes’ D patients, respectively. In both subgroups, *RASSF1* methylation was associated with a worse OS (HR 3.06 95% CI 1.27–7.50; HR 5.76 95% CI 2.44–14.82, respectively) [106].

For *CDKN2A* (*p14)* methylation, the study of Nilsson et al. suggests a worse prognosis in TNM stages I–IV (*p* = 0.036) [105], while the studies of Cleven et al. and Chaar et al. do not confirm these findings [73, 99]. Subgroup analyses also do not show a statistically significant influence of *CDKN2A* (*p14)* methylation on prognosis [73].

Hypermethylation of *APC* was reported to be positively associated with survival by Chen et al. (HR 0.426 95% CI 0.190–0.957) [43]; however, two independent studies did not confirm these results [73, 105]. In contrast, in a subgroup of 66 stage II MSS and *BRAF* wildtype patients, *APC* methylation was associated with worse cancer-specific survival (HR 2.63 95% CI 1.21–5.68) [73]. Matthaios et al. investigated *APC* methylation in Dukes’ A–C and in Dukes’ D patients, respectively. In both subgroups, *APC* methylation was associated with a worse OS (HR 7.88 95% CI 2.73–22.73; HR 3.47 95% CI 1.35–8.92, respectively) [106].

Methylation of *CHFR* as a prognostic marker was assessed in only two independent studies. In the study by Tanaka et al., *CHFR* methylation was associated with poorer RFS in TNM stage I–IV patients (HR 2.99 95% CI 1.23–7.23) [72]. This was not confirmed by the study of Cleven et al., which reported no association with CSS in TNM stage I–IV patients (HR 1.33 95% CI 0.81–2.20) [73]. Subgroup analysis for TNM stage II and III patients was done in both studies. The study of Tanaka et al. reported no association between *CHFR* methylation and RFS in TNM stage II patients (HR 1.45 95% CI 0.30–6.88), but a significant association between *CHFR* methylation and RFS in TNM stage III patients (HR 4.31 95% CI 1.34–12.8) [72]. In the study of Cleven et al. subgroup analyses of TNM stage II, MSS and *BRAF*wt patients showed a significant association between *CHFR* methylation and CSS (HR 3.89 95% CI 1.58–9.60), but results were not validated in an independent patient series. Subgroup analysis of TNM stage III, MSS, and *BRAF*wt patients did not show a significant association with prognosis [73].

*SEPT9* was assessed as a biomarker in two studies. Liu et al. did not find a statistically significant association between methylation and prognosis in TNM stage I–IV or TNM stage I–III patients [109]. The study of Tham et al. reports an association between *SEPT9* methylation and worse OS in TNM stage I–III patients (HR 3.50 95% CI 1.67–7.32) [108]. Although they appeared to have assessed *SEPT9* methylation as a biomarker, the study of Perez-Carbonell did not give any specific information on the outcome of this analysis [90].

For three markers (*ID4*, *MYOD1*, and *SFRP2*) and two marker panels (*AXIN2* & *DKK1* and *CDKN2A & hMLH1*), analyses were performed in two or more independent studies or patient populations, but reported results were too limited to construct a forest plot (Additional file 6). Methylation of *ID4* was assessed by two studies. Umetani et al. reported a significant association between *ID4* methylation in stage I–IV CRC patients and poorer OS (HR 1.82 95% CI 1.09–3.43) [88], but the study of Tanaka et al. did not confirm this (*p* = 0.118) [72]. *MYOD1* was suggested as a prognostic biomarker in the study of Hiranuma et al. (HR 3.16 95% CI 1.25–8.02) [97], but this was not seen in the study of Shannon et al. (*p* = 0.14) [96]. Also, for *SFRP2* methylation, survival data were only reported in one out of two studies [70, 111]. Tang et al. observed a statistically significant association with OS in stage I–IV CRC patients (HR 3.06 95% CI 1.12–8.40) [111].

Of 83 included studies, 20 studies assessed the prognostic influence of multimarker panels. Although the same markers were included in several multimarker panels, only three panels were assessed in two or more independent studies or patient populations. For one panel consisting of *MLH1* and *CDKN2A,* a better prognosis in TNM stage I–IV patients was reported in the study of Veganzones et al. (*p* = 0.04) [66]. However, in the study by Aoyagi et al.*,* methylation of both genes was associated with worse survival in TNM stage IV patients (*p* = 0.03; Additional file 6) [85]. The study of Gaedcke et al. described a panel of markers (*ADAP1*, *BARHL2*, *CABLES2*, *DOT1L*, *ERAS*, *ESRG*, *RNF220*, *ST6GALNAC5*, *TAF4*, *SLC20A2*) that was associated with poorer DFS in two independent study series (HR 3.57 95% CI 1.01–12.55; HR 3.78 95% CI 1.26–11.37; Fig. 2p) [40]. Kandimalla et al. studied another panel, combing the markers *AXIN2* and *DKK1* (Additional file 6), in two independent TNM stage II populations (*n* = 65 and *n* = 79, respectively). In both populations, an association with poorer RFS was found (HR 3.84 95% CI 1.14–12.43; *p* < 0.0004, respectively) [45].

### Clinical translation

For a definite conclusion on validity of a (prognostic) biomarker, a sufficient level of evidence (LoE) is needed. Methylation marker results (i.e., similar conclusions drawn in two or more independent study series) were ranked according to two established ranking schemes to obtain a comprehensive summary of the current evidence on prognostic epigenetic biomarkers in CRC [36, 37]. For 12 single markers, and two multimarker panels, consistent results were reported in two or more publications or populations (Table 1). For four markers, the current LoE is level II, and for the other markers, LoE is lower. For 11 other markers and one multimarker panel, reported results are still too inconclusive to draw any conclusion on a possible prognostic biomarker effect (Table 1).

**Table 1:** Level of evidence (LoE) of methylation markers assessed in two or more study series

Marker	Population	Reported association	LoE	REMARK score Median (range)^c^
Consistent results^a^				
IGFBP3	TNM stages 2–3	Poor prognosis	II	13.8 (11–14)
CDKN2A (p16)	TNM stages 1–3^b^	Poor prognosis	II	11.5 (6.5–17.5)
WNT5a	TNM stages 1–4	No influence on prognosis	II	12.5 (−)
HPP1	TNM stage 4	Poor prognosis	II	12.5 (10–15)
RET	TNM stage 2	Poor prognosis	II–III	15.5 (−)
TFPA2E	TNM stages 1–4	Good prognosis	III	7.8 (5–10.5)
Multimarker panel by Gaedcke et al. [40]	Locally advanced rectal cancer	Poor prognosis	III	12 (−)
Multimarker panel by Kandimalla et al. [45]	TNM stage II	Poor prognosis	III	15
HLTF	TNM stages 1–4	Poor prognosis	III–IV	15 (10–17.5)
HPP1	TNM stages 1–4	Poor prognosis	IV	12.5 (10–15)
EVL	TNM stages 1–4	Poor prognosis	IV	11
CD109	TNM stages 1–4	Poor prognosis	IV	11
NRCAM	TNM stages 1–4	Poor prognosis	IV	11
FLNC	TNM stages 1–4	Poor prognosis	IV	11
Inconclusive results				
BNIP3				11.5 (17.5–9)
MLH1				11.3 (10–15)
MGMT				12 (5–17.5)
RASSF1A				10.5 (5–17.5)
CDKN2A (p14)				8 (5–17.5)
APC				8.5 (5–17.5)
CHFR				16 (17.5–14.5)
ID4				9.5 (−)
MYOD1				9.5 (9–10)
SEPT9				14.75 (14.5–15)
SFRP2				11 (10.5–11.5)
MLH1 & CDKN2A				11 (10–12)

^a^≥ 2 studies in similar populations showing consistent results

^b^“Summarized definition”; subgroup definitions differ between specific studies

^c^All studies included that contribute to LoE, or all overall results if LoE was inconclusive

## Discussion

In this review, we summarized published studies on prognostic DNA methylation markers for CRC. Although a large number of studies were identified and included in this review, the results from individual studies are difficult to compare due to the variation in study design, methodology, and survival endpoints. The number of prognostic biomarkers that were considered in multiple independent studies or patient populations is low, and promising results observed in one study are often not validated in another.

In 2005, with the publication of the REMARK guidelines, an attempt was made to improve the reporting quality of biomarker studies [25]. However, the observed variation in reporting sample series characteristics, statistical analyses, and sample sizes indicates that the REMARK guidelines are still not completely adapted and that accurate reporting of prognostic DNA methylation markers needs improvement (median REMARK score 10.7 out of 20). As we observed that studies which reported significant findings had lower REMARK scores (Fig. 3a, *p* = 0.005) than studies which did not, a stricter adherence to the REMARK guidelines might be helpful, if we ever want to draw definitive conclusions on the role of a possible biomarker [27]. A more rigorous peer review by the scientific journals might be justified, in order to achieve this [123]. However, the REMARK guidelines are open to subjective interpretation, just as the scoring of the REMARK criteria. An inadequately reported methylation marker study does not imply that the methylation marker itself is not valuable, but it might hinder reproducibility.

The observed inconsistencies in individual study results might have various reasons, such as differences in sample collection, sample preparation, methods of DNA methylation analysis, and the genomic location of the assay [22, 124–126]. The lack of standardization of different methods is a major issue in DNA methylation research. Differences in one or more technical aspects of the detection method used, including primer design, reagents, equipment, and protocols, can result in different DNA methylation measurements, even for the exact same genomic location, and can therefore have a substantial impact on the prognostic value of a test [127, 128]. Therefore, even consistently reported methylation marker results from this review should be treated carefully and validated in powered prospective studies. Comparing the results of methylation markers obtained with different methodology was not within the scope of this review, however should be addressed in a meta-analysis of the markers with most evidence.

The same lack of standardization holds true for the statistical analysis and the choice of study endpoints [129, 130]. Whereas many studies focus on overall survival, other studies use cause-specific, disease-specific, or recurrence-free survival or do not specify the endpoints that were considered. As there are no uniform definitions of these endpoints, it is difficult to compare individual study results [34].

It is generally accepted that CRC is a heterogeneous disease with diverse subgroups, both on histological and molecular level [131–136]. Analyzing all CRC patients as one group will therefore obscure the true potential of some biomarkers. The majority of studies in this review (68 studies; 82%) performed an analysis in TNM stages I–IV. As to date, TNM stage is one of the most important prognostic factors in cancer and the choice to include all TNM stages in one analysis will most likely influence the final conclusion. To overcome this, most studies also included one or more subgroup analyses, e.g., subgroups based on MSI, KRAS, or TNM stage. However, the definition of these subgroups is often very detailed or specific, thereby hampering the comparability of individual study results [38]. For example, 13 different subgroup analyses were identified for *CDKN2A* (p16) in this systematic review that could not be combined in a meta-analysis. In addition, different subgroups may have different baseline risks of death, thereby even further hindering comparison between different studies. Thus, on the one hand, analyzing CRC as a homogenous group could hinder the discovery of subgroup-specific biomarkers; however, on the other hand, analyzing subgroups that are too specific hinders the possibility of validation or meta-research.

Although the risk of introducing selection bias when selecting patients solely based on the availability of tissue is recognized [137], most studies in this review were retrospective (67 studies; 81%) and conducted in a small number of patients (64 studies included < 200 patients; 77%). Often, those study populations are often patient series that have been collected in research laboratories and University Hospitals based on the availability of samples. This, in addition to the availability of follow-up data, often determines the size of the study population. This approach however does not sufficiently contribute to answering the question whether a biomarker had prognostic value and if it should be implemented to improve patient care. It has already been shown that the prognostic effect of DNA methylation markers assessed in small sample series are often chance findings that cannot be reproduced in independent series [138, 139]. Also in this review, we observed that statistically significant *p* values tend to be reported more often in studies with a small population size (Fig. 3c). In order to increase the LoE of prognostic methylation markers, large-scale prospectively collected study populations are required. Therefore, we need a more structured approach, with collaborations between research groups, to obtain sufficient numbers of patient samples and validation populations to draw final conclusions on the prognostic relevance of a biomarker [140]. Twenty-three single markers and two multimarker panels have been investigated in two or more independent studies or patient populations. For eight biomarkers (*IGFBP3*, *CDKN2A (p16*), *WNT5a*, *HPP1*, *RET*, *TFPA2E*, *HLTF, CD109, NRCAM, FLNC*, and *EVL*) and the marker panels proposed by Gaedcke et al. (*ADAP1*, *BARHL2*, *CABLES2*, *DOT1L*, *ERAS*, *ESRG*, *RNF220*, *ST6GALNAC5*, *TAF4*, *SLC20A2*) and Kandimalla et al. (*AXIN2*, *DKK1*), the results were consistent and a statistically significant association with prognosis was observed despite differences in study design and methodology. For five biomarkers (*IGFBP3*, *CDKN2A (p16)*, *WNT5a*, *HPP1*, and *RET*), the current LoE was II–III, indicating that a definitive conclusion on the prognostic influence of these markers is within reach. However, before a definitive conclusion on these markers can be made, a large, prospective study aimed at studying the clinical validity or a meta-analysis of studies with LoE II, which will be difficult given the differences in study design and methodology, is needed. Solely for *WNT5a*, additional studies assessing the prognostic influence might be omitted as results show no prognostic influence with a current LoE of II. For the other markers (*TFPA2E*, *HLTF*, *HPP1*, and the multimarker panel proposed by Gaedcke et al.), current LoE is greater than or equal to III, indicating that even more validation is needed. To increase the LoE, these validation studies should preferably be prospectively designed, aimed at studying the biomarker effect, instead of retrospective case-series as these contribute little to increasing the LoE.

Reporting according to guidelines such as REMARK is important but not sufficient for successful translation of prognostic DNA methylation markers to clinical practice. Promising prognostic DNA methylation markers should be evaluated in multivariable prediction models to study their added prognostic value compared to the current reference standard (TNM stage) and other novel strategies to predict CRC prognosis [141–143]. Few of the included prognostic methylation marker studies in this review have assessed the incremental value of their prognostic DNA methylation marker in addition to the golden standard TNM staging system or other suggested prognostic markers such as grade of differentiation or microsatellite instability (MSI) [144]. In addition, other potential prognostic tools, such as histologic and molecular markers [145, 146], the immunoscore [147], circulating tumor DNA (ctDNA) [148], or the consensus molecular subtype (CMS) classification [149] should be taken into account in prediction models, as it is likely that a combination of several different types of markers will eventually yield the best predictive power.

## Conclusion

Despite the widespread acceptance of epigenetic alterations as possible important biomarkers for CRC prognosis, very few biomarkers reach the point of usability in daily patient care and comprehensive overviews of the abundantly available biomarker results are lacking. In this review, we identified several promising markers that all require different amounts of further validation before definitive conclusions on their clinical applicability can be drawn. We also identified multiple problems hampering the comparison of individual study results including problems with population selection, study design, technical issues, and validation problems. Adhering to the REMARK guidelines might partly overcome these problems, and a more rigorous peer-review process specifically focusing on these reporting issues might be an essential step towards reducing the number of chance findings. In addition, biomarker research would benefit from a more structured approach in multidisciplinary collaborations, including clinicians, epidemiologists, statisticians, technicians, and molecular biologists, aiming to perform large, well-designed, and validated biomarker studies [140]. Only then will we be able to ultimately assess the clinical value of a biomarker.

## Additional files

Additional file 1: **Table S1.** Search terms used for systematic review. (DOCX 83 kb)

Additional file 2: **Table S2.** REMARK checklist and description for scoring the reviewed studies. (DOCX 94 kb)

Additional file 3: **Table S3.** Characteristics of 83 included studies. (XLSX 95 kb)

Additional file 4: **Table S4.** Scoring of 83 included studies according to REMARK. (DOCX 114 kb)

Additional file 5: **Table S5.** Risk of potential bias and confounders of the included studies. Studies indicated by a “X” potentially have an increased risk of bias, whereas studies indicated by a “√” potentially have a decreased risk of bias. (DOCX 108 kb)

Additional file 6: **Table S6.** Single markers and their characteristics that have been investigated in more than one study series, however, of which Cox regression survival analysis was not available for all markers. (DOCX 63 kb)
