# Supplementary material for: Prognostic DNA methylation markers for sporadic colorectal cancer: a systematic review
Source: Clin Epigenetics. 2018 Mar 14;10:35. doi: 10.1186/s13148-018-0461-8 (PMC5851322; doi:10.1186/s13148-018-0461-8)
Supplement: Supplementary file 1 — Table S1. Search terms used for systematic review. (DOCX 83 kb) [file 13148_2018_461_MOESM1_ESM.docx]

Table S1. Search terms used for systematic review.

|  | PubMed | EMBASE | Medline |
| --- | --- | --- | --- |
| 1 | "methylation"[MeSH Terms] OR "methylation"[All Fields] OR "dna methylation"[MeSH Terms] OR "dna"[All Fields] AND "methylation"[All Fields] OR "dna methylation"[All Fields] OR "methylation"[MeSH Terms] OR "methylation"[All Fields] OR hypermethylation[All Fields] OR hypomethylation[All Fields] OR promoter[All Fields] AND "methylation"[MeSH Terms] OR "methylation"[All Fields] | ‘DNA methylation’ OR ‘hypermethylation’ OR ‘hypomethylation’ OR ‘methylation’ OR ‘promoter methylation’ | ‘DNA methylation’ OR ‘hypermethylation’ OR ‘hypomethylation’ OR ‘methylation’ OR ‘promoter methylation’ |
| 2 | "colorectal neoplasms"[MeSH Terms] OR "colorectal"[All Fields] AND "neoplasms"[All Fields] OR "colorectal neoplasms"[All Fields] OR "colorectal"[All Fields] AND "cancer"[All Fields] OR "colorectal cancer"[All Fields] OR "colonic neoplasms"[MeSH Terms] OR "colonic"[All Fields] AND "neoplasms"[All Fields] OR "colonic neoplasms"[All Fields] OR "colon"[All Fields] AND "cancer"[All Fields] OR "colon cancer"[All Fields] | ‘colorectal cancer’ OR ‘colon cancer’ OR ‘rectal cancer’ | ‘colorectal cancer’ OR ‘colon cancer’ OR ‘rectal cancer’ |
| 3 | "prognosis"[MeSH Terms] OR "prognosis"[All Fields] OR "mortality"[Subheading] OR "mortality"[All Fields] OR "survival"[All Fields] OR "survival"[MeSH Terms] OR "patients"[MeSH Terms] OR "patients"[All Fields] OR "patient"[All Fields] AND outcome[All Fields] | ‘prognosis’ OR ‘patient outcome’ OR ‘survival’ OR ‘survival’ | ‘prognosis’ OR ‘patient outcome’ OR ‘survival’ OR ‘survival’ |
| Search | 1 AND 2 AND 3 | 1 AND 2 AND 3 | 1 AND 2 AND 3 |
