# Supplementary material for: Prognostic DNA methylation markers for sporadic colorectal cancer: a systematic review
Source: Clin Epigenetics. 2018 Mar 14;10:35. doi: 10.1186/s13148-018-0461-8 (PMC5851322; doi:10.1186/s13148-018-0461-8)
Supplement: Supplementary file 2 — Table S2. REMARK checklist and description for scoring the reviewed studies. (DOCX 94 kb) [file 13148_2018_461_MOESM2_ESM.docx]

Table S2. REMARK checklist [34] and description for scoring the reviewed studies.

| REMARK checklist item | Description | Our interpretation and consequence for scoring |
| --- | --- | --- |
| Introduction |  |  |
| 1 | State the marker examined, study objectives and pre-specified hypotheses. |  |
| Materials and Methods |  |  |
| Patients |  |  |
| 2 | Describe the characteristics (eg disease stage or co-morbidities) of study patients, including their source and inclusion and exclusion criteria | Co-morbidities were never mentioned, therefore we did not include this aspect in our analysis |
| 3 | Describe treatments received and how chosen (eg randomized or rule-based). | If information was provided, whether or whether not treatment was provided, study was positively scored. |
| Specimen characteristics |  |  |
| 4 | Describe the type of biological material used (incl. control samples) and methods for preservation. |  |
| Assay methods |  |  |
| 5 | Specify the assay method used and provide (or reference) a detailed protocol, incl. specific reagents or kits used, quality control procedures, reproducibility assessment, quantitation methods, and scoring and reporting protocols. Specify whether and how assays were performed blinded to the study endpoint. |  |
| Study design |  |  |
| 6 | State the method of case selection, including whether prospective or retrospective and whether stratification or matching (eg by stage of disease or age) was used. Specify the time period from which cases were taken, the end of the follow-up period, and the median-follow-up time. | When not explicitly stated in the article, we considered a study as retrospective, if the time period was stated. |
| 7 | Precisely define all clinical endpoints examined. |  |
| 8 | List all candidate variables initially examined or considered for inclusion in models. |  |
| 9 | Give rational for sample size; if the study was designed to detect a specified effect size, give the target power and effect size. | We considered a rational, if the authors of the study mentioned the number of patients with follow-up data |
| Statistical analysis methods |  |  |
| 10 | Specify all statistical methods, including details of any variable selection procedures and other model-building issues, how model assumptions were verified, and how missing data were handled. |  |
| 11 | Clarify how marker values were handled in the analyses; if relevant, describe methods used for cutpoint determination. |  |
| Results |  |  |
| Data |  |  |
| 12 | Describe the flow of patients through the study, including the number of patients included in each stage of the analysis (a diagram may be helpful) and reasons for dropout. Specifically, both overall and for each subgroup extensively examined report the number of patients and the number of events. |  |
| 13 | Report distributions of basic demographic characteristics (at least age and sex), standard (disease-specific) prognostic variables, and tumor marker, including number of missing values. |  |
| Analysis and interpretation |  |  |
| 14 | Show the relation of the marker to standard prognostic variables |  |
| 15 | Present univariable analysis showing the relation between the marker and outcome, with the estimated effect (eg hazard ratio and survival probability). Preferably provide similar analyses for all other variables being analyzed. For the effect of a tumor marker on a time-to-event outcome, a Kaplan-Meier plot is recommended. |  |
| 16 | For key multivariable analyses, report estimated effects (eg hazard ratio) with confidence intervals for the marker and, at least for the final model, all other variables in the model. |  |
| 17 | Among reported results, provide estimated effects with confidence intervals from an analysis in which the marker and standard prognostic variables are included, regardless of their statistical significance. |  |
| 18 | If done, report results of further investigations, such as checking assumptions, sensitivity analysis, and internal validation. | We scored studies which addressed one of the aspects with 1 point |
| Discussion |  |  |
| 19 | Interpret the results in the context of the pre-specified hypotheses and other relevant studies; include a discussion of limitations of the study. |  |
| 20 | Discuss implications for further research and clinical value. |  |
