# Supplementary material for: Prognostic DNA methylation markers for sporadic colorectal cancer: a systematic review
Source: Clin Epigenetics. 2018 Mar 14;10:35. doi: 10.1186/s13148-018-0461-8 (PMC5851322; doi:10.1186/s13148-018-0461-8)
Supplement: Supplementary file 4 — Table S4. Scoring of 83 included studies according to REMARK. (DOCX 114 kb) [file 13148_2018_461_MOESM4_ESM.docx]

Table S4. Scoring of 83 included studies according to REMARK.

|  |  | Marker description | Patient characteristics | Information about treatment | Biological material & preservation method | Assay method | Sample selection & follow-up time | Clinical endpoint definition | Variables included in analysis | Rational for sample size | Specification of statistical methods | Handling of marker values | Description of flow of patients through study | Basic demographic characteristics | Relation marker to standard variables | Univariable analysis | Multivariable analysis | Marker & standard variables | Further investigations (eg internal validation) | Interpretations of results & limitations of study | Implications for further research | Score |
| --- | --- | --- | --- | --- | --- | --- | --- | --- | --- | --- | --- | --- | --- | --- | --- | --- | --- | --- | --- | --- | --- | --- |
| REMARK item |  | 1 | 2 | 3 | 4 | 5 | 6 | 7 | 8 | 9 | 10 | 11 | 12 | 13 | 14 | 15 | 16 | 17 | 18 | 19 | 20 | SCORE |
| Marker | Study |  |  |  |  |  |  |  |  |  |  |  |  |  |  |  |  |  |  |  |  |  |
| ADAP1, BARHL2, CABLES2, DOT1L, ERAS, ESRRG, RNF220, ST6GALNAC5, TAF4, SLC20A2 | Gaedcke et al. 2014 | 1 | 1 | 1 | 1 | 0.5 | 0.5 | 1 | 0.5 | 0 | 0.5 | 0 | 0.5 | 1 | 0 | 1 | 0 | 0 | 0.5 | 1 | 1 | 12 |
| AOX-1, RARB2, RERG, ADAMTS9, IRF4, FOXE-1 | Luo et al. 2016 | 1 | 1 | 0 | 1 | 1 | 0.5 | 1 | 0 | 0 | 1 | 0.5 | 0 | 1 | 0 | 1 | 1 | 0 | 0 | 1 | 0.5 | 11.5 |
| Apaf-1, BCL2, p53 | Benard et al. 2015 | 1 | 1 | 1 | 1 | 1 | 1 | 0.5 | 0 | 0.5 | 0.5 | 0 | 0.5 | 1 | 1 | 1 | 1 | 0.5 | 1 | 0.5 | 0.5 | 14.5 |
| APC, MGMT | Chen et al. 2009 | 1 | 0.5 | 1 | 0.5 | 0.5 | 0.5 | 0.5 | 0 | 0 | 0.5 | 0 | 0.5 | 1 | 0 | 0 | 1 | 0 | 0 | 0.5 | 0.5 | 8.5 |
| ASCL2, APCDD1, AXIN2, DKK1, LGR5 | De Sousa et al. 2011 | 0.5 | 0.5 | 0 | 0 | 0.5 | 0.5 | 0 | 0 | 0 | 0.5 | 0 | 0.5 | 0.5 | 0 | 0.5 | 0 | 0 | 0.5 | 0.5 | 0.5 | 5.5 |
| AXIN2, DKK1, APCDD1, ASCL2 | Kandimalla et al. 2017 | 1 | 1 | 1 | 1 | 1 | 0.5 | 1 | 1 | 0 | 0.5 | 0.5 | 0 | 1 | 0 | 1 | 1 | 0.5 | 1 | 1 | 1 | 15 |
| BMP6 | Sangplod et al. 2014 | 1 | 0.5 | 0 | 1 | 1 | 0.5 | 0.5 | 0 | 0 | 0 | 0 | 0 | 0.5 | 1 | 0 | 0 | 0 | 0 | 0.5 | 0.5 | 7 |
| BNIP3 | Shimizu et al. 2010 | 1 | 0.5 | 1 | 1 | 1 | 0 | 0 | 0 | 0 | 0.5 | 0 | 0.5 | 0 | 0.5 | 1 | 1 | 0.5 | 0 | 0.5 | 0 | 9 |
| CD109, EVL, FLNC, NRCAM, IGFBP3 | Yi et al. 2011 | 0.5 | 0.5 | 1 | 1 | 1 | 1 | 0 | 0 | 0 | 0.5 | 0 | 0.5 | 1 | 0.5 | 0.5 | 0.5 | 0.5 | 1 | 0.5 | 0.5 | 11 |
| CDH13, FLBN3, DLEC1, hMLH1, RUNX3 | Wang et al. 2012 | 1 | 0.5 | 1 | 1 | 1 | 1 | 0 | 0 | 0 | 0.5 | 0 | 0.5 | 0.5 | 0.5 | 0.5 | 0.5 | 1 | 0 | 0.5 | 0 | 10 |
| CDKN2A (p16) | Liang et al. 1999 | 1 | 1 | 1 | 1 | 1 | 1 | 0 | 0 | 0 | 0.5 | 0 | 0.5 | 0.5 | 0.5 | 0.5 | 0 | 0 | 0 | 0.5 | 0.5 | 9.5 |
| CDKN2A (p16) | Esteller et al. 2009 | 1 | 1 | 0.5 | 0.5 | 0.5 | 1 | 0 | 0 | 0.5 | 0.5 | 0 | 0.5 | 0.5 | 0 | 0.5 | 0.5 | 0 | 0 | 0.5 | 0.5 | 9 |
| CDKN2A (p16) | Maeda et al. 2003 | 1 | 0 | 0 | 0.5 | 0.5 | 0.5 | 0 | 0 | 0 | 0.5 | 0 | 0.5 | 0.5 | 0.5 | 0.5 | 0 | 0 | 0 | 0 | 0.5 | 6.5 |
| CDKN2A (p16) | Sanz-Casla et al. 2005 | 1 | 1 | 1 | 1 | 1 | 1 | 0 | 0 | 0 | 0.5 | 0 | 0.5 | 0.5 | 0.5 | 0.5 | 0 | 0 | 0 | 0.5 | 0.5 | 9.5 |
| CDKN2A (p16) | Nakayama et al. 2007 | 1 | 0 | 0 | 0.5 | 1 | 0 | 0 | 0 | 0 | 0.5 | 0 | 0.5 | 1 | 1 | 1 | 0 | 0 | 0 | 0.5 | 0 | 7 |
| CDKN2A (p16) | Wettergren et al. 2008 | 1 | 0.5 | 1 | 1 | 1 | 0.5 | 1 | 0 | 0.5 | 0.5 | 0 | 0.5 | 1 | 1 | 0.5 | 1 | 0 | 0 | 0.5 | 0 | 11.5 |
| CDKN2A (p16) | Wettergren et al. 2010 | 1 | 1 | 0.5 | 0.5 | 0.5 | 1 | 1 | 0 | 0 | 0.5 | 0 | 0.5 | 1 | 0 | 1 | 1 | 0.5 | 0 | 0.5 | 0.5 | 11 |
| CDKN2A (p16) | Malhotra et al. 2010 | 1 | 0.5 | 0.5 | 1 | 0.5 | 1 | 0.5 | 0 | 0 | 0.5 | 0 | 0 | 0 | 0 | 1 | 0 | 0 | 0 | 1 | 0 | 7.5 |
| CDKN2A (p16) | Mitomi et al. 2009 | 1 | 1 | 1 | 1 | 1 | 0.5 | 1 | 0 | 0.5 | 0.5 | 0 | 0.5 | 0.5 | 1 | 1 | 1 | 1 | 0 | 0.5 | 0.5 | 13.5 |
| CDKN2A (p16) | Shima et al. 2011 | 1 | 0.5 | 0.5 | 1 | 0.5 | 1 | 1 | 1 | 0 | 1 | 0 | 0.5 | 1 | 1 | 1 | 1 | 1 | 1 | 1 | 1 | 16 |
| CDKN2A (p16) | Bihl et al. 2012 | 1 | 0.5 | 0 | 0.5 | 0.5 | 0.5 | 0 | 0 | 0 | 0.5 | 0 | 0.5 | 0.5 | 1 | 0.5 | 1 | 1 | 0 | 0.5 | 0.5 | 9 |
| CDKN2A (p16) | Veganzones-de-Castro et al. 2012 | 1 | 1 | 1 | 1 | 1 | 0.5 | 1 | 0 | 0.5 | 0.5 | 0 | 0.5 | 1 | 1 | 1 | 0 | 0.5 | 0 | 0.5 | 0.5 | 12.5 |
| CDKN2A (p16) | Kohonen-Corish et al. 2014 | 1 | 1 | 0.5 | 1 | 0.5 | 1 | 1 | 0 | 0 | 0.5 | 0 | 0.5 | 1 | 0 | 1 | 1 | 0.5 | 0 | 1 | 0.5 | 12 |
| CDKN2A (p16), p15 | Ishiguro et al. 2006 | 1 | 0.5 | 0 | 1 | 1 | 0 | 1 | 0 | 0 | 0.5 | 0 | 0 | 1 | 1 | 0 | 0 | 0 | 0 | 1 | 1 | 9 |
| CDKN2A (p16), hMLH1 | Aoyagi et al. 2011 | 1 | 1 | 1 | 1 | 0.5 | 0.5 | 0 | 0 | 0 | 0.5 | 0 | 0.5 | 1 | 1 | 1 | 0 | 0 | 0 | 0.5 | 0.5 | 10 |
| CDKN2A (p16), hMLH1 | Miladi-Abdennadher et al. 2011 | 1 | 0.5 | 1 | 1 | 1 | 0.5 | 0.5 | 0 | 0 | 0.5 | 0 | 0.5 | 1 | 1 | 1 | 1 | 0 | 0 | 1 | 0.5 | 12 |
| CDKN2A (p16), BNIP3, hMLH1 | Iida et al. 2012 | 1 | 1 | 1 | 1 | 1 | 1 | 0 | 0 | 0 | 0.5 | 0 | 0.5 | 0.5 | 0.5 | 0.5 | 1 | 1 | 0 | 0.5 | 0.5 | 11.5 |
| CDKN2A (p16), hMLH1 | Veganzones et al. 2015 | 1 | 1 | 1 | 1 | 0.5 | 0.5 | 1 | 0.5 | 0 | 0.5 | 0 | 0.5 | 1 | 1 | 1 | 0 | 0 | 0 | 1 | 0.5 | 12 |
| CDKN2A (p16), MGMT | Krtolica et al. 2007 | 1 | 0.5 | 1 | 1 | 1 | 0.5 | 0.5 | 0 | 0 | 0.5 | 0 | 0.5 | 0 | 1 | 1 | 0 | 0 | 0 | 0.5 | 0.5 | 9.5 |
| CDKN2A (p16), APC, CDH1, MGMT | Kamiyama et al. 2009 | 0.5 | 0.5 | 1 | 1 | 1 | 0.5 | 0 | 0 | 0.5 | 0.5 | 0 | 0.5 | 0 | 1 | 0.5 | 0 | 0 | 0 | 0.5 | 0.5 | 8.5 |
| CDKN2A (p16), hMLH1, MGMT | Kuan et al. 2015 | 1 | 0.5 | 0 | 0.5 | 1 | 0.5 | 1 | 1 | 0 | 0.5 | 0 | 0.5 | 1 | 0.5 | 0.5 | 0.5 | 0.5 | 0 | 1 | 0.5 | 11 |
| CDKN2A (p16), IGFBP7, KLOTHO, hMLH1,PHD3, SFRP2 | Yang et al. 2014 | 1 | 0.5 | 0.5 | 1 | 1 | 0 | 0 | 1 | 0 | 0.5 | 0 | 0.5 | 1 | 1 | 0 | 1 | 0.5 | 0 | 0.5 | 0.5 | 10.5 |
| CDX2 | Jiang et al. 2016 | 1 | 1 | 0.5 | 0.5 | 1 | 0.5 | 0 | 0 | 0 | 0.5 | 0 | 0.5 | 0.5 | 1 | 0.5 | 1 | 0 | 0 | 0.5 | 0.5 | 9.5 |
| CHFR, ID4, RECK, MINT1 | Tanaka et al. 2011 | 1 | 1 | 0.5 | 1 | 1 | 0.5 | 0.5 | 1 | 1 | 0.5 | 0 | 0.5 | 0.5 | 1 | 0 | 1 | 1 | 0.5 | 1 | 1 | 14.5 |
| CHFR, PLOD1, CDKN2A (p16), CDKN2A (p14ARF), MGMT, RASSF1, APC, HLTF, GATA4, GATA5, ADAM23, RAB32, JPH3, FOXL2, BNIP3, NEURL1, CACNA2, THBS1, TFPI2 | Cleven et al. 2014 | 1 | 1 | 1 | 1 | 1 | 1 | 1 | 1 | 0.5 | 0.5 | 0 | 0.5 | 1 | 1 | 1 | 1 | 1 | 1 | 1 | 1 | 17.5 |
| DACT2 | Wang et al. 2015 | 1 | 0.5 | 0 | 0.5 | 0.5 | 0 | 0 | 0 | 0 | 0.5 | 0 | 0.5 | 0 | 0.5 | 0.5 | 1 | 0.5 | 0 | 0.5 | 0.5 | 7 |
| DSC3 | Cui et al. 2011 | 1 | 0.5 | 1 | 0.5 | 1 | 0 | 0 | 0 | 0 | 0.5 | 0 | 0.5 | 0.5 | 0.5 | 0.5 | 0 | 0 | 0.5 | 0.5 | 0.5 | 8 |
| EPAS1 | Rawlusko-Wieczorek et al. 2014 | 1 | 1 | 1 | 1 | 1 | 0.5 | 1 | 0 | 1 | 0.5 | 0 | 0.5 | 1 | 0 | 0 | 1 | 0.5 | 0 | 0.5 | 0.5 | 12 |
| FBLN1 | Xu et al. 2015 | 1 | 0.5 | 0.5 | 0.5 | 1 | 0.5 | 1 | 0.5 | 0 | 0.5 | 0 | 0 | 1 | 1 | 0.5 | 0 | 0.5 | 0 | 0.5 | 0 | 9.5 |
| HLTF, HPP1, hMLH1 | Wallner et al. 2006 | 1 | 0.5 | 0.5 | 1 | 1 | 1 | 1 | 1 | 1 | 0.5 | 0 | 0.5 | 1 | 1 | 1 | 1 | 1 | 0 | 0.5 | 0.5 | 15 |
| HLTF, HPP1 | Herbst et al. 2009 | 1 | 0.5 | 0 | 1 | 1 | 1 | 1 | 1 | 1 | 0.5 | 0 | 0.5 | 1 | 1 | 1 | 1 | 1 | 0 | 0.5 | 0.5 | 14.5 |
| HLTF, HPP1 | Philipp et al. 2012 | 1 | 0 | 1 | 1 | 1 | 0 | 1 | 0 | 1 | 0.5 | 0 | 0.5 | 1 | 1 | 0.5 | 0.5 | 0.5 | 0.5 | 0.5 | 1 | 12.5 |
| HLTF, HPP1 | Philipp et al. 2014 | 1 | 0.5 | 1 | 1 | 1 | 0 | 0.5 | 0 | 0 | 0.5 | 0 | 0.5 | 1 | 1 | 0.5 | 0.5 | 0 | 0 | 0.5 | 0.5 | 10 |
| HPP1 | Herbst et al. 2017 | 1 | 1 | 1 | 1 | 1 | 0.5 | 1 | 0 | 0.5 | 1 | 1 | 0.5 | 1 | 0 | 1 | 0.5 | 0.5 | 0.5 | 1 | 1 | 15 |
| hMLH1 | Jensen et al. 2013 | 1 | 0.5 | 0 | 1 | 1 | 0 | 1 | 1 | 0 | 0.5 | 0 | 0.5 | 1 | 0 | 1 | 0 | 0 | 0.5 | 1 | 1 | 11 |
| hMLH1 | Wang et al. 2014 | 1 | 0.5 | 0.5 | 1 | 0.5 | 0 | 0.5 | 0 | 0 | 0.5 | 0 | 0.5 | 1 | 1 | 1 | 0.5 | 0 | 0 | 1 | 0.5 | 10 |
| hMLH1, hMSH2 | Malhotra et al. 2014 | 1 | 0.5 | 0.5 | 1 | 1 | 0.5 | 0.5 | 0 | 0 | 0.5 | 0 | 0 | 0 | 0 | 0 | 0 | 0 | 0 | 0.5 | 0.5 | 6.5 |
| HOPX | Katoh et al. 2012 | 1 | 0.5 | 0 | 0.5 | 1 | 0.5 | 0 | 0 | 0 | 0.5 | 0 | 0.5 | 1 | 0.5 | 1 | 1 | 0.5 | 0 | 0.5 | 0.5 | 9.5 |
| ID4 | Umetani et al. 2004 | 1 | 0.5 | 0 | 0.5 | 1 | 0.5 | 0 | 0 | 0.5 | 0.5 | 0 | 0.5 | 0 | 1 | 1 | 1 | 1 | 0 | 0.5 | 0 | 9.5 |
| IGFBP3 | Fu et al. 2015 | 1 | 0.5 | 0.5 | 1 | 1 | 1 | 0 | 0 | 0 | 0.5 | 0 | 0.5 | 1 | 1 | 1 | 1 | 1 | 1 | 1 | 1 | 14 |
| IGFBP3, ALX4, GAS7 | Perez-Carbonell et al. 2014 | 1 | 1 | 0.5 | 0.5 | 1 | 0.5 | 1 | 1 | 0 | 0.5 | 0 | 0 | 1 | 1 | 1 | 1 | 1 | 0 | 1 | 0.5 | 13.5 |
| IGF2 | Baba et al. 2010 | 1 | 0.5 | 0.5 | 1 | 1 | 1 | 1 | 1 | 0.5 | 1 | 0.5 | 0.5 | 1 | 1 | 1 | 1 | 1 | 0.5 | 1 | 1 | 17 |
| KISS-1 | Moya et al. 2013 | 1 | 0 | 0 | 1 | 0.5 | 0 | 1 | 0 | 0 | 0.5 | 0 | 0.5 | 1 | 0.5 | 1 | 0 | 0 | 1 | 0.5 | 0.5 | 9 |
| LGR5 | Su et al. 2015 | 1 | 1 | 1 | 0.5 | 1 | 0.5 | 0 | 0.5 | 0 | 0.5 | 0 | 0 | 1 | 0.5 | 0 | 0 | 0 | 0.5 | 0.5 | 0.5 | 9 |
| MGMT | Shima et al. 2011 | 1 | 0.5 | 0.5 | 1 | 0.5 | 1 | 1 | 1 | 0.5 | 0.5 | 0.5 | 0.5 | 1 | 1 | 1 | 1 | 0.5 | 1 | 1 | 1 | 16 |
| MGMT | Oliver et al. 2014 | 1 | 1 | 1 | 1 | 1 | 1 | 0.5 | 0.5 | 0 | 0.5 | 0 | 0.5 | 1 | 0 | 1 | 0.5 | 0.5 | 0 | 0.5 | 0.5 | 12 |
| MYOD1 (Myf-3) | Shannon et al. 1999 | 1 | 0 | 0 | 0.5 | 0.5 | 0 | 0.5 | 0 | 0 | 0.5 | 0 | 0 | 0 | 0.5 | 0.5 | 0 | 0 | 0 | 0.5 | 0 | 4.5 |
| MYOD1 | Hiranuma et al. 2004 | 1 | 0.5 | 0 | 1 | 1 | 0.5 | 0 | 0 | 0 | 0.5 | 0 | 0.5 | 1 | 1 | 0 | 1 | 0 | 0 | 0.5 | 0.5 | 9 |
| SYNPO2 (Myopodin) | Esteban et al. 2012 | 1 | 0 | 0 | 1 | 0.5 | 0 | 1 | 1 | 0 | 0.5 | 0 | 0.5 | 0.5 | 1 | 0.5 | 0 | 0 | 1 | 0.5 | 1 | 10 |
| CDKN2A (p14/ARF) | Chaar et al. 2014 | 1 | 0.5 | 0 | 1 | 1 | 0 | 0 | 0 | 0 | 0.5 | 0 | 0.5 | 0.5 | 1 | 1 | 0 | 0 | 0 | 0.5 | 0.5 | 8 |
| PCDH10, SPARC, UCHL1, UCHL2, UCHL3, FZD9, SFRP1, WIF1 | Heitzer et al. 2014 | 1 | 0.5 | 1 | 1 | 1 | 1 | 1 | 1 | 0.5 | 0.5 | 0 | 1 | 0.5 | 1 | 1 | 0 | 0 | 0.5 | 0.5 | 1 | 14 |
| PPARG | Pancione et al. 2010 | 1 | 0.5 | 1 | 1 | 1 | 1 | 0.5 | 0 | 0 | 0.5 | 0 | 0 | 1 | 1 | 1 | 0 | 0 | 0.5 | 0.5 | 0.5 | 11 |
| PTEN | Lin et al. 2015 | 1 | 0.5 | 1 | 1 | 1 | 1 | 1 | 0 | 0 | 0.5 | 0 | 0.5 | 1 | 1 | 1 | 1 | 0.5 | 0.5 | 0.5 | 0.5 | 13.5 |
| RARß2, RASSF1, CDH1, DABK1 | Miladi-Abdennadher et al. 2010 | 1 | 0.5 | 1 | 0.5 | 1 | 0.5 | 0 | 0 | 0.5 | 0.5 | 0 | 0.5 | 0.5 | 1 | 1 | 0.5 | 0.5 | 0 | 1 | 0.5 | 11 |
| RASSF1 (RASSF1A) | Chen et al. 2012 | 1 | 0.5 | 1 | 0.5 | 1 | 0.5 | 0.5 | 0 | 0 | 0.5 | 0 | 0.5 | 1 | 0 | 1 | 1 | 0 | 0 | 0.5 | 0.5 | 10 |
| RASSF1 (RASSF1A), CDKN2A (p14/ARF), APC1A | Nilsson et al. 2013 | 1 | 0.5 | 0 | 0 | 0.5 | 0 | 0 | 0 | 0 | 0.5 | 0 | 0.5 | 0 | 0.5 | 0 | 0.5 | 0 | 0 | 0.5 | 0.5 | 5 |
| RASSF1 (RASSF1A), APC | Matthaios et al. 2016 | 1 | 0.5 | 0 | 1 | 1 | 0.5 | 0.5 | 0 | 0 | 0.5 | 0.5 | 0 | 1 | 1 | 0.5 | 0.5 | 0 | 0 | 0.5 | 0.5 | 9.5 |
| RET | Draht et al. 2014 | 1 | 1 | 1 | 1 | 1 | 0.5 | 1 | 1 | 0.5 | 0.5 | 0 | 0.5 | 0 | 1 | 1 | 1 | 0.5 | 1 | 1 | 1 | 15.5 |
| TAC1, SEPT9, NELL1 | Tham et al. 2015 | 1 | 1 | 1 | 1 | 0.5 | 1 | 1 | 0 | 0 | 0.5 | 0 | 0.5 | 1 | 0 | 1 | 1 | 1 | 1 | 1 | 1 | 14.5 |
| TAC1, SEPT9, NELL1, EYA4, CRABP1, MAL, SST | Liu et al. 2016 | 1 | 1 | 1 | 1 | 0.5 | 1 | 1 | 0 | 0.5 | 0.5 | 0.5 | 0.5 | 1 | 0.5 | 1 | 1 | 0.5 | 0.5 | 1 | 1 | 15 |
| SFRP1, SLIT2, HIC1, MYOD1, RASSF1, APC, MGMT | Dallol et al. 2012 | 1 | 0.5 | 0 | 1 | 1 | 0.5 | 1 | 0 | 0.5 | 0.5 | 0 | 0.5 | 1 | 0.5 | 1 | 0.5 | 0 | 0 | 0.5 | 0 | 10 |
| SFRP2 | Tang et al. 2011 | 1 | 0.5 | 1 | 1 | 0.5 | 0 | 0 | 0 | 0.5 | 0 | 0 | 0.5 | 1 | 1 | 0 | 1 | 1 | 0 | 0.5 | 0.5 | 10 |
| SHISA3 | Tsai et al. 2015 | 1 | 0.5 | 1 | 1 | 0.5 | 1 | 1 | 1 | 1 | 0.5 | 0 | 0.5 | 1 | 1 | 0.5 | 0.5 | 0.5 | 0 | 0.5 | 0 | 13 |
| SLFN11 | He et al. 2017 | 1 | 0.5 | 0 | 0.5 | 1 | 0.5 | 0.5 | 0 | 0 | 0.5 | 0 | 0 | 1 | 1 | 1 | 1 | 0.5 | 0.5 | 0.5 | 0.5 | 10.5 |
| SOCS-1 | Kang et al. 2016 | 1 | 0.5 | 1 | 1 | 0.5 | 0 | 0 | 0 | 0 | 0.5 | 0.5 | 0 | 1 | 1 | 0.5 | 0 | 0 | 0 | 0.5 | 0.5 | 8.5 |
| SYK (Syk) | Yang et al. 2013 | 1 | 0 | 0 | 0.5 | 1 | 0 | 1 | 0 | 0 | 0.5 | 0 | 0.5 | 0 | 1 | 0.5 | 1 | 0 | 0 | 0.5 | 0 | 7.5 |
| TBX5 | Yu et al. 2010 | 1 | 0 | 0 | 0.5 | 1 | 0 | 0 | 0 | 0.5 | 0.5 | 0 | 0.5 | 0 | 1 | 1 | 1 | 1 | 0 | 0.5 | 0.5 | 9 |
| TFAP2E | Zhang et al. 2014 | 1 | 0.5 | 0.5 | 1 | 1 | 0 | 0.5 | 0 | 0 | 0.5 | 0 | 0.5 | 1 | 1 | 1 | 0.5 | 0 | 0 | 1 | 0.5 | 10.5 |
| TFAP2E | Park et al. 2015 | 1 | 1 | 1 | 0.5 | 0.5 | 0.5 | 1 | 1 | 0 | 0.5 | 0 | 0.5 | 1 | 1 | 1 | 1 | 0.5 | 0.5 | 1 | 0.5 | 14 |
| TFAP2E | Beggs et al. 2015 | 1 | 0 | 0 | 0 | 0.5 | 0 | 0.5 | 0.5 | 0 | 0.5 | 0 | 0 | 0 | 0 | 1 | 0 | 0 | 0 | 0.5 | 0.5 | 5 |
| TWIST1 | Ruppenthal et al. 2011 | 1 | 0.5 | 0.5 | 1 | 1 | 0.5 | 1 | 0 | 0 | 0.5 | 0 | 0.5 | 1 | 1 | 0 | 0 | 0 | 0 | 0.5 | 0.5 | 9.5 |
| WIF-1 | Abdelmaksoud-Dammak et al. 2014 | 1 | 1 | 1 | 1 | 1 | 0 | 0 | 0 | 0 | 0.5 | 0 | 0 | 1 | 1 | 1 | 0 | 0 | 0 | 0.5 | 0 | 9 |
| Wnt5a | Rawson et al. 2011 | 1 | 0.5 | 0 | 1 | 1 | 1 | 1 | 0 | 0 | 0.5 | 0 | 0.5 | 1 | 1 | 0.5 | 1 | 0.5 | 1 | 0.5 | 0.5 | 12.5 |
|  |  |  |  |  |  |  |  |  |  |  |  |  |  |  |  |  |  |  |  |  |  |  |
