# Supplementary material for: Prognostic DNA methylation markers for sporadic colorectal cancer: a systematic review
Source: Clin Epigenetics. 2018 Mar 14;10:35. doi: 10.1186/s13148-018-0461-8 (PMC5851322; doi:10.1186/s13148-018-0461-8)
Supplement: Supplementary file 5 — Table S5. Risk of potential bias and confounders of the included studies. Studies indicated by a “X” potentially have an increased risk of bias, whereas studies indicated by a “√” potentially have a decreased risk of bias. (DOCX 108 kb) [file 13148_2018_461_MOESM5_ESM.docx]

Table S5. Risk of potential bias and confounders of the included studies. Studies indicated by a “X“ potentially have an increased risk of bias, whereas studies indicated by a “√“ potentially have a decreased risk of bias.

|  | Selection bias | Assay method  (measurement bias) | Outcome assessment (measurement bias) | Confounders | REMARK Score |
| --- | --- | --- | --- | --- | --- |
| **Study** |  |  |  |  |  |
| Gaedcke et al. 2014 | **√** | **X** | **√** | **X** | 12 |
| Luo et al. 2016 | **√** | **√** | **√** | **√** | 11.5 |
| Benard et al. 2015 | **√** | **X** | **X** | **√** | 14.5 |
| Chen et al. 2009 | **X** | **X** | **X** | **√** | 8.5 |
| De Sousa et al. 2011 | **X** | **X** | **X** | **X** | 5.5 |
| Kandimalla et al. 2017 | **√** | **√** | **√** | **√** | 15 |
| Sangplod et al. 2014 | **X** | **X** | **X** | **X** | 7 |
| Shimizu et al. 2010 | **X** | **X** | **X** | **√** | 9 |
| Yi et al. 2011 | **√** | **X** | **X** | **X** | 11 |
| Wang et al. 2012 | **√** | **X** | **X** | **X** | 10 |
| Liang et al. 1999 | **√** | **X** | **X** | **X** | 9.5 |
| Esteller et al. 2009 | **√** | **X** | **X** | **X** | 9 |
| Maeda et al. 2003 | **X** | **X** | **X** | **X** | 6.5 |
| Sanz-Casla et al. 2005 | **√** | **X** | **X** | **X** | 9.5 |
| Nakayama et al. 2007 | **X** | **X** | **X** | **X** | 7 |
| Wettergren et al. 2008 | **X** | **X** | **√** | **√** | 11.5 |
| Wettergren et al. 2010 | **√** | **X** | **√** | **√** | 11 |
| Malhotra et al. 2010 | **√** | **X** | **X** | **X** | 7.5 |
| Mitomi et al. 2009 | **√** | **X** | **√** | **√** | 13.5 |
| Shima et al. 2011 | **√** | **X** | **√** | **√** | 16 |
| Bihl et al. 2012 | **X** | **X** | **X** | **√** | 9 |
| Veganzones-de-Castro et al. 2012 | **√** | **X** | **√** | **X** | 12.5 |
| Kohonen-Corish et al. 2014 | **√** | **X** | **√** | **√** | 12 |
| Ishiguro et al. 2006 | **X** | **X** | **√** | **X** | 9 |
| Aoyagi et al. 2011 | **√** | **X** | **X** | **X** | 10 |
| Miladi-Abdennadher et al. 2011 | **X** | **X** | **X** | **√** | 12 |
| Iida et al. 2012 | **√** | **X** | **X** | **√** | 11.5 |
| Veganzones et al. 2015 | **√** | **X** | **√** | **X** | 12 |
| Krtolica et al. 2007 | **X** | **X** | **X** | **X** | 9.5 |
| Kamiyama et al. 2009 | **X** | **X** | **X** | **X** | 8.5 |
| Kuan et al. 2015 | **X** | **X** | **√** | **X** | 11 |
| Yang et al. 2014 | **X** | **X** | **X** | **√** | 10.5 |
| Jiang et al. 2016 | **√** | **X** | **X** | **√** | 9.5 |
| Tanaka et al. 2011 | **√** | **X** | **X** | **√** | 14.5 |
| Cleven et al. 2014 | **√** | **X** | **√** | **√** | 17.5 |
| Wang et al. 2015 | **X** | **X** | **X** | **√** | 7 |
| Cui et al. 2011 | **X** | **X** | **X** | **X** | 8 |
| Rawlusko-Wieczorek et al. 2014 | **√** | **X** | **√** | **√** | 12 |
| Xu et al. 2015 | **X** | **X** | **√** | **X** | 9.5 |
| Wallner et al. 2006 | **√** | **X** | **√** | **√** | 15 |
| Herbst et al. 2009 | **√** | **X** | **√** | **√** | 14.5 |
| Philipp et al. 2012 | **X** | **X** | **√** | **X** | 12.5 |
| Philipp et al. 2014 | **X** | **X** | **X** | **X** | 10 |
| Herbst et al. 2017 | **√** | **√** | **√** | **√** | 15 |
| Jensen et al. 2013 | **X** | **X** | **√** | **X** | 11 |
| Wang et al. 2014 | **X** | **X** | **X** | **X** | 10 |
| Malhotra et al. 2014 | **X** | **X** | **X** | **X** | 6.5 |
| Katoh et al. 2012 | **X** | **X** | **X** | **√** | 9.5 |
| Umetani et al. 2004 | **X** | **X** | **X** | **√** | 9.5 |
| Fu et al. 2015 | **√** | **X** | **X** | **√** | 14 |
| Perez-Carbonell et al. 2014 | **√** | **X** | **√** | **√** | 13.5 |
| Baba et al. 2010 | **√** | **√** | **√** | **√** | 17 |
| Moya et al. 2013 | **X** | **X** | **√** | **X** | 9 |
| Su et al. 2015 | **√** | **X** | **X** | **X** | 9 |
| Shima et al. 2011 | **√** | **√** | **√** | **√** | 16 |
| Oliver et al. 2014 | **√** | **X** | **X** | **X** | 12 |
| Shannon et al. 1999 | **X** | **X** | **X** | **X** | 4.5 |
| Hiranuma et al. 2004 | **X** | **X** | **X** | **√** | 9 |
| Esteban et al. 2012 | **X** | **X** | **√** | **X** | 10 |
| Chaar et al. 2014 | **X** | **X** | **X** | **X** | 8 |
| Heitzer et al. 2014 | **√** | **X** | **√** | **X** | 14 |
| Pancione et al. 2010 | **√** | **X** | **X** | **X** | 11 |
| Lin et al. 2015 | **√** | **X** | **√** | **√** | 13.5 |
| Miladi-Abdennadher et al. 2010 | **X** | **X** | **X** | **√** | 11 |
| Chen et al. 2012 | **X** | **X** | **X** | **√** | 10 |
| Nilsson et al. 2013 | **X** | **X** | **X** | **X** | 5 |
| Matthaios et al. 2016 | **X** | **√** | **X** | **X** | 9.5 |
| Draht et al. 2014 | **√** | **X** | **√** | **√** | 15.5 |
| Tham et al. 2015 | **√** | **X** | **√** | **√** | 14.5 |
| Liu et al. 2016 | **√** | **X** | **√** | **√** | 15 |
| Dallol et al. 2012 | **X** | **X** | **√** | **X** | 10 |
| Tang et al. 2011 | **X** | **X** | **X** | **√** | 10 |
| Tsai et al. 2015 | **√** | **X** | **√** | **X** | 13 |
| He et al. 2017 | **X** | **X** | **X** | **√** | 10.5 |
| Kang et al. 2016 | **X** | **X** | **X** | **X** | 8.5 |
| Yang et al. 2013 | **X** | **X** | **√** | **√** | 7.5 |
| Yu et al. 2010 | **X** | **X** | **X** | **√** | 9 |
| Zhang et al. 2014 | **X** | **X** | **X** | **X** | 10.5 |
| Park et al. 2015 | **√** | **X** | **√** | **√** | 14 |
| Beggs et al. 2015 | **X** | **X** | **X** | **X** | 5 |
| Ruppenthal et al. 2011 | **X** | **X** | **√** | **X** | 9.5 |
| Abdelmaksoud-Dammak et al. 2014 | **X** | **X** | **X** | **X** | 9 |
| Rawson et al. 2011 | **√** | **X** | **√** | **√** | 12.5 |
