# Supplementary material for: Prognostic DNA methylation markers for sporadic colorectal cancer: a systematic review
Source: Clin Epigenetics. 2018 Mar 14;10:35. doi: 10.1186/s13148-018-0461-8 (PMC5851322; doi:10.1186/s13148-018-0461-8)
Supplement: Supplementary file 6 — Table S6. Single markers and their characteristics that have been investigated in more than one study series, however, of which Cox regression survival analysis was not available for all markers. (DOCX 63 kb) [file 13148_2018_461_MOESM6_ESM.docx]

Table S6. Single markers and their characteristics that have been investigated in more than one study series, however of which Cox regression survival analysis was not available for all markers.

| Study | Marker | Endpoint | HR (95% CI) | P | N | Stage | REMARK score |
| --- | --- | --- | --- | --- | --- | --- | --- |
| Kandimalla et al. 2017 | AXIN2, DKK1 | RFS | 3.84 (1.14-12.43) |  | 65 | II | 15 |
| Kandimalla et al. 2017 | AXIN2, DKK1 | RFS |  | <0.0004 | 79 | II | 15 |
| Aoyagi et al. 2011 | CDKN2A, MLH1 | OS |  | 0.0318 | 134 | IV | 10 |
| Veganzones et al. 2015 | CDKN2A, MLH1 | DFS | 0.028 (0.0-42.1) | 0.04 | 51 | I-IV | 12 |
| Umetani et al. 2004 | ID4 | OS | 1.82 (1.09-3.43) |  | 76 | I-IV | 9.5 |
| Tanaka et al. 2011 | ID4 | OS |  | 0.118 | 82 | II-III | 14.5 |
| Shannon et al. 1999 | MYOD1 (Myf-3) | NA |  | 0.14 | NA | II-III | 4.5 |
| Hiranuma et al. 2004 | MYOD1 | OS | 3.16 (1.25-8.02) |  | 80 | II-III | 9 |
| Tang et al. 2011 | SFRP2 | OS | 3.06 (1.12-8.40) |  | 77 | I-IV | 10 |
| Yang et al. 2014 | SFRP2 | Mortality | NA | NA | 184 | I-IV | 10.5 |

NA = not reported

DFS = Disease-free survival

OS = Overall survival

HR = Hazard ratio Cox regression analysis

P = p-value Kaplan-Meier
